# Interpretability of Deep High-Frequency Residuals: A Case Study on SAR Splicing Localization

**DOI:** 10.3390/jimaging11100338

**Published:** 2025-09-28

**Authors:** Edoardo Daniele Cannas, Sara Mandelli, Paolo Bestagini, Stefano Tubaro

**Affiliations:** Image and Sound Processing Lab (ISPL), Dipartimento di Elettronica, Informazione e Bioingegneria, Politecnico di Milano, Via Ponzio 34/5, 20133 Milan, Italy; edoardodaniele.cannas@polimi.it (E.D.C.); paolo.bestagini@polimi.it (P.B.); stefano.tubaro@polimi.it (S.T.)

**Keywords:** Multimedia Forensics, Deep High-Frequency Residuals (DHFRs), image splicing localization, interpretability, SAR, explainability, xAI

## Abstract

Multimedia Forensics (MMF) investigates techniques to automatically assess the integrity of multimedia content, e.g., images, videos, or audio clips. Data-driven methodologies like Neural Networks (NNs) represent the state of the art in the field. Despite their efficacy, NNs are often considered “black boxes” due to their lack of transparency, which limits their usage in critical applications. In this work, we assess the interpretability properties of Deep High-Frequency Residuals (DHFRs), i.e., noise residuals extracted from images by NNs for forensic purposes, that nowadays represent a powerful tool for image splicing localization. Our research demonstrates that DHFRs not only serve as a visual aid in identifying manipulated regions in the image but also reveal the nature of the editing techniques applied to tamper with the sample under analysis. Through extensive experimentation on spliced amplitude Synthetic Aperture Radar (SAR) images, we establish a correlation between the appearance of the DHFRs in the tampered-with zones and their high-frequency energy content. Our findings suggest that, despite the deep learning nature of DHFRs, they possess significant interpretability properties, encouraging further exploration in other forensic applications.

## 1. Introduction

Multimedia Forensics (MMF) focuses on assessing the integrity of multimedia objects, such as digital pictures, audio clips, videos, or satellite imagery [1,2]. Historically, researchers looked for forensic footprints, i.e., traces left by editing operations at a signal processing level to unveil the history of the digital object at hand. However, data-driven solutions like Neural Networks (NNs) and Convolutional Neural Networks (CNNs) now represent the state of the art in the field [3]. NNs can automatically extract meaningful features from data corpora, lifting the burden on researchers to look for specific footprints, and are nowadays outperforming standard signal processing techniques on almost every forensic task.

One main criticism against data-driven techniques is their lack of interpretability and explainability [4,5,6]. NNs are often regarded as “black box” tools, meaning that it is unclear what elements in their input are important for making a decision or how NNs process them [6]. These properties are relevant whenever data-driven tools make high-stakes choices, e.g., in criminal justice or healthcare domains. The same applies to MMF, whose relevance in law, justice, and fighting misinformation is paramount [7,8].

Over the years, high-frequency residuals extracted from images by CNNs, here defined for simplicity “Deep High-Frequency Residuals (DHFRs)”, have been demonstrated to be a powerful instrument for MMF, especially for image splicing localization [9,10,11]. This task focuses on spatially localizing traces left by editing operations locally applied to the image, e.g., inserting a portion of an image into another one or deleting a pixel area from the sample under attack. State-of-the-art solutions based on DHFRs, like the Noiseprint [10], the TruFor [11], and the detector developed in [9], provide a heatmap localizing spliced areas as inconsistencies in the editing traces of the image under analysis. This heatmap can be inspected by the naked eye, giving immediate feedback to the user about the clues found by the CNN. Therefore, we can assert to a certain extent that such solutions are interpretable.

This work aims to dig deeper into the interpretability capabilities of DHFRs. We claim not only that DHFRs provide visual feedback but that the appearance of the tampered-with area indicates the nature of the editing executed on it.

We focus on the specific scenario of amplitude Synthetic Aperture Radar (SAR) image splicing localization for our experiments. SAR images are bi-dimensional representations of backscattered radar waves with numerous applications, especially in intelligence and military operations [12,13,14,15,16]. Spliced amplitude SAR images are images in which a malicious actor has substituted a pixel region with another one coming from a different sample, and additional processing has been applied to hinder this manipulation [9]. SAR images are characterized by phenomena like speckle noise, layover, or multi-path effects [13]. Moreover, their generation process involves many signal processing operations, e.g., resampling [17], ground-range projection [18], etc. To make a spliced sample more plausible, a malicious user can resort to a wider range of editing operations with respect to natural images, such as noise addition, blurring, resizing, etc. All these operations have different impacts on the high-frequency residual extracted from these images, making SAR imagery splicing localization particularly fascinating for our study.

**Figure 1:**
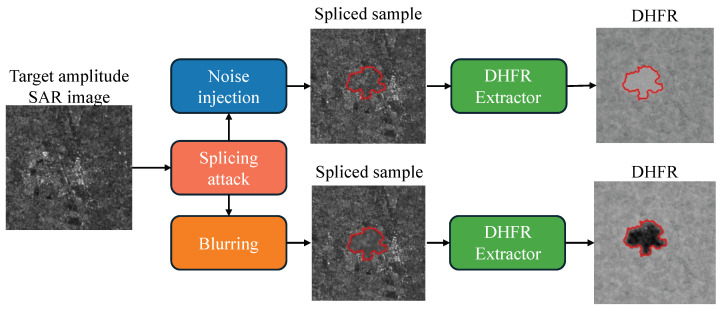
A schematic illustration of the proposed investigations on the interpretability of DHFRs in the context of amplitude SAR image splicing localization. In our experiments, we show how the appearance of DHFRs (extracted by state-of-the-art detectors working on SAR amplitude data [9]) depends on the nature of the editing operations executed on the spliced region (indicated with a red contour in the figure).

Figure 1 reports an example of our findings. We show that whenever a malicious actor applies a blurring or a resampling edit on the tampered-with area, the resulting pixels in the DHFR present low amplitude, i.e., they appear as “dark spots”; conversely, whenever the user applies noise-based editing, the tampered-with area appears as a “bright” spot. We conjecture this behavior is related to the nature of DHFRs and to the considered editing operations: blur-like editing works as low-pass filtering, hence diminishing the power of the signal in the high-frequency range and resulting in low-brightness pixels; noise-based editing increases the power of the signal and widens its spectral content, enhancing the DHFR brightness.

We evaluate our findings on a dataset of spliced amplitude SAR images presented in [9], confirming that DHFRs of blurring and noise-based edited samples appear consistently across the whole dataset. Moreover, we experimentally check our previously reported intuition, i.e., that the appearance of spliced areas in the DHFR links to their power spectral content.

The main contributions of our work are the following:

- We show that DHFRs extracted from spliced amplitude SAR images present different appearance depending on the nature of the editing operation executed on them;
- We link this phenomenon to the ability of DHFR to capture high-frequency-related traces, in particular, the energy content of the image in the high-frequency range.

## 2. Background

### 2.1. Multimedia Forensics and High-Pass Frequency Residuals

Historically, MMF focused on analyzing digital pictures. Stamm et al. [19] provide a detailed overview of all the techniques and tasks undertaken in recent years. For instance, many works propose techniques to detect Forensic Footprints (FFs) left by operations executed on the whole picture, e.g., resampling operations [20,21,22], application of median filters [23,24], or multiple image compressions [25,26,27].

Another lively research topic is the localization of splicing editing. As mentioned earlier, splicing refers to inserting a portion of an image into another one, with the possible execution of further editing, aiming to conceal a specific pixel area. Splicing localization means spatially identifying (i.e., at a pixel level) which areas of an image are under editing. Many works in the literature rely on the information carried by the so-called noise or high-frequency residual to accomplish this task [28,29,30]. This residual is a picture obtained by removing the high-level semantic content from the image through high-pass filtering, assuming that editing traces leave distinctive marks in high-frequency ranges.

Thanks to the automatic extraction of forensic traces executed by data-driven methods, deep learning approaches have become popular in MMF. CNNs in particular are now the State Of The Art (SOTA) for the task of image tampering localization. Some works combine CNNs with the idea of high-frequency residuals by either using a fixed high-pass convolutional filter as the first layer of the network [31,32] or making the network learn the most appropriate filter during training [33,34].

More recent contributions further elaborate on this idea by exploiting the representation capabilities of Denoiser-Convolutional Neural Networks (Dn-CNNs), i.e., CNNs developed for image denoising. Its basic functioning consists of estimating a DHFR from the image under analysis. In particular, the forensic community heavily exploited the Dn-CNN [35], e.g., for PRNU anonymization [36]. This is also the case with Noiseprint [10], i.e., a Dn-CNN that extracts a noise residual, suppressing the vast majority of the image content, and exposes editing-related artifacts due to local image forgery. When analyzing pristine images, the Noiseprint is self-consistent, whereas in the case of spliced images, it highlights the edited regions. Table 1 reports an overview of all solutions exploiting the DHFR and its applications.

### 2.2. SAR Imagery and Forensics

Due to the diffusion of portals that offer them for free, in recent years, satellite images have been targeted by malicious manipulations [1], including splicing editing [38,39]. Among the different modalities of imagery available, Synthetic Aperture Radar (SAR) refers to a particular modality of satellite data consisting of bi-dimensional representations of backscattered radar waves. Such data has a wide range of applications [40] thanks to its independence from cloud coverage, weather conditions, and daylight [12,13,41]. These characteristics are particularly appealing in intelligence and military operations, i.e., to detect sensible military targets like airports [42] and aircrafts [14], ships [15], tanks, or other vehicles [16].

SAR images are affected by some characteristic phenomena, like layover, speckle noise, etc., that, together with their peculiar lifecycle, make standard FFs not suitable for their analysis [1]. However, especially when provided as amplitude products, they are easy to manipulate with standard image editing software like Photoshop or GIMP [9], or even synthetic image manipulation tools [1,43].

For these reasons and their centrality in sensitive applications, the forensic community developed tailored solutions. An example is [9], where the authors adapted the concept of DHFR to the SAR scenario to localize splicing editing on amplitude imagery. Similarly to [10], their core idea is to train a Dn-CNN to extract consistent DHFR only for pristine amplitude SAR tiles, i.e., whenever an SAR image is spliced, the tampered-with area should present with a different appearance due to the inhomogeneity of local processing traces. The authors then tested their solution against a dataset of spliced amplitude SAR images, showing good localization performances across various editing operations applied to the spliced area.

## 3. Amplitude SAR Imagery Splicing Localization

The lifecycle of SAR data is characterized by processing pipelines and degradation phenomena that vary from simple resampling procedures to different kinds of noises [9,43]. A malicious actor realizing a splicing attack may rely on various editing operations to make it more plausible, from noise additions and speckle noise injection to blurring or affine transformations. All these operations have different impacts on the frequency content of the tampered-with area, making amplitude SAR imagery splicing localization a relevant case study.

Formally, we define a spliced amplitude SAR image as follows. Let us consider two amplitude SAR images, a donor image D and a target T. A splicing operation modifies the pixels in a target region T of T using pixels from a donor region D of D. As explained previously, a malicious user might also apply some editing to make the splicing more plausible. Without loss of generalization, we hypothesize the user first creates an edited version of the donor image E(D), with E(·) being a suitable editing function, and then selects D accordingly. Notice that T and D have the same shape and orientation but might differ for their position in T and E(D), respectively. The final spliced image S is defined as [9]

(1) $$S\left(x,y\right)=\left\{\begin{array}{cc} D(x^{\prime },y^{\prime }) & \;\mathrm{if}\;(x,y)\in T\\ T(x,y), & \;\mathrm{if}\;(x,y)\notin T \end{array}\right.,$$

with $\left(x^{\prime },y^{\prime }\right)$ being the point coordinates of the donor region corresponding to the target coordinate system (x,y).

We can define the integrity of the spliced sample S with a tampering mask M, taking values 0 or 1 depending on the pixels being pristine or spliced. More formally [9],

(2) $$M\left(x,y\right)=\left\{\begin{array}{c} 1,\;\mathrm{if}\;(x,y)\in T\\ 0,\;\mathrm{if}\;(x,y)\notin T \end{array}\right..$$

In [9], the authors have recently proposed a forensic detector for localizing splicing areas on amplitude SAR images. This detector highlights local inconsistencies between the donor and target pixel areas and proves superior to state-of-the-art tools developed for natural pictures. Its functioning is based on a denoising CNN [35] which extracts a real-valued fingerprint map, i.e., a Deep High-Frequency Residual (DHFR), exposing local inconsistencies between pixels. Formally, we define the DHFR extracted from the spliced image as [9]

(3) R=f(S),

where f(·) represents the detector operator, while R is a real-valued matrix with the same resolution of the input image. We can consider the function f(·) as a high-pass filtering operator that captures traces relative to the processing pipeline of the considered image. As a result, the DHFR highlights spliced pixels as inconsistencies in these traces [9].

## 4. SAR DHFR Interpretability Analysis

As mentioned previously, the discriminative power of high-frequency residuals rely on their capability to highlight local processing traces. Indeed, many editing operations that commonly affect photographs, such as resampling or compression, leave peculiar artifacts in the high-frequency range that become visible after a simple high-pass filtering [28,29,30]. We argue that this property is also true for DHFRs extracted from amplitude SAR images and that we can obtain further insight into the nature of the editing operation executed by simple visual inspection.

### 4.1. Experimental Setup

For our investigations, we rely on a portion of the dataset presented in [9] and available at https://github.com/polimi-ispl/dhfr_interpretability (accessed on 22 September 2025). The selected dataset is defined as Spliced Dataset 2 (SD2), which comprises around 7000 spliced amplitude SAR images with various editing operations modifying the manipulated area, i.e., Additive White Gaussian Noise (AWGN), Additive Laplacian Noise (ALN), Speckle Noise (SN) injection, Average Blur (AB), Median Blur (MB), and affine transforms such as random Rotation & Resize (R&R). We also consider the case where no additional editing has been executed on the spliced region.

As for the forensic detector, we rely on the Augmented SAR Adapted Extractor (ASAE), which is the best performing localization model presented in [9]. We use this detector as it is from our original work, without retraining or fine-tuning it (please refer to [9] for more details).

### 4.2. DHFR Visual Inspection

**Figure 2:**
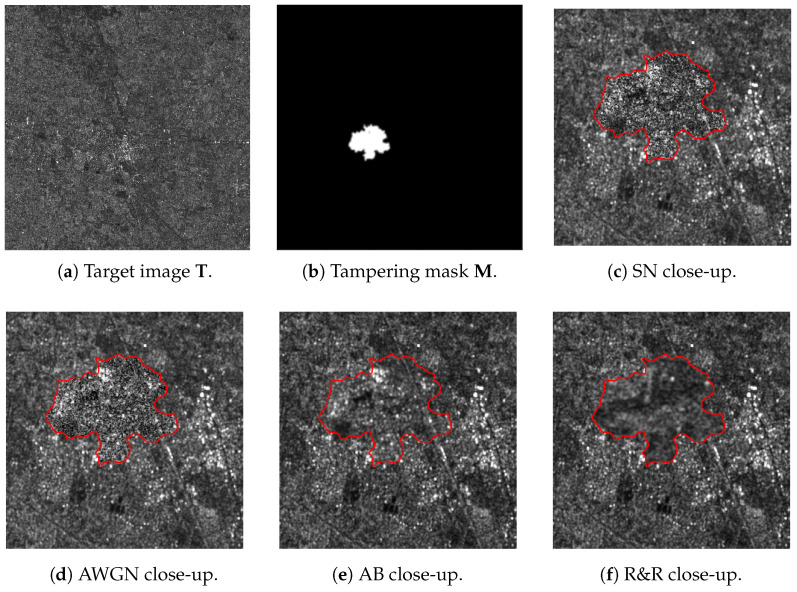
Four different examples of spliced images. We provide the target image T in the first row, first column, and the tampering mask M in the first row, second column. The first row, third column, and second row, second column show a close-up around the manipulated area edited with SN and AWGN. On the last figures, i.e., second row, second and third columns, the editing is AB and R&R. Once again, we highlight the spliced area with a red contour.

**Figure 3:**
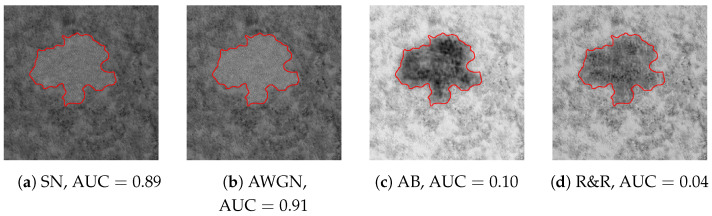
DHFRs extracted from the spliced samples of Figure 2. Once again, the spliced area is highlighted with red contours. (**a**,**b**) present different brightness values in the spliced area with respect to (**c**,**d**). This difference is also reflected in the AUC values, as explained in Section 4.

After extracting the DHFR from all the images in SD2, we first visually inspect a few of them and compare the effects of different editing. Figure 2 reports four examples of spliced images undergoing diverse editing, while the DHFRs extracted by the ASAE detector are shown in Figure 3. In particular, in Figure 3a,b, we report the DHFRs obtained from amplitude images spliced using SN and AWGN (noise-based manipulations) as editing operations. In Figure 3c,d, the operations are instead AB and R&R (blur-based manipulations). We can immediately notice that the first two columns’ DHFRs present bright pixels in the manipulated area, while the last two columns show a dark spot.

### 4.3. Consistency Across the Dataset

We perform two additional investigations to confirm our visual intuition, that is, bright spots occur in DHFRs extracted from images spliced with noise-based and dark spots in case of blur-based editing.

As a first analysis, we extract the pixels in correspondence with the manipulated area from each DHFR. We then compute the Multi-Dimensional Scaling (MDS) [44] from them. MDS is a dimensionality reduction technique, i.e., a technique to visualize high-dimensional data points such as vectors. Roughly speaking, if two data points are close in their MDS representation, their original Euclidean distance is small, i.e., they are adjacent in their high-dimensional definition space.

**Figure 4:**
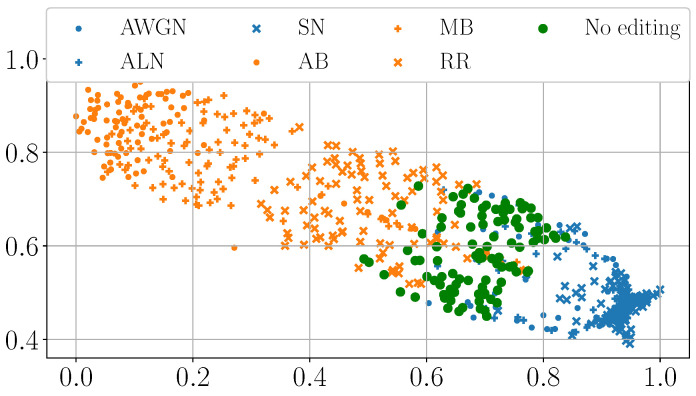
MDS visualization of the pixels belonging to the tampered-with area extracted from the DHFRs of 128×128 spliced samples.

**Figure 5:**
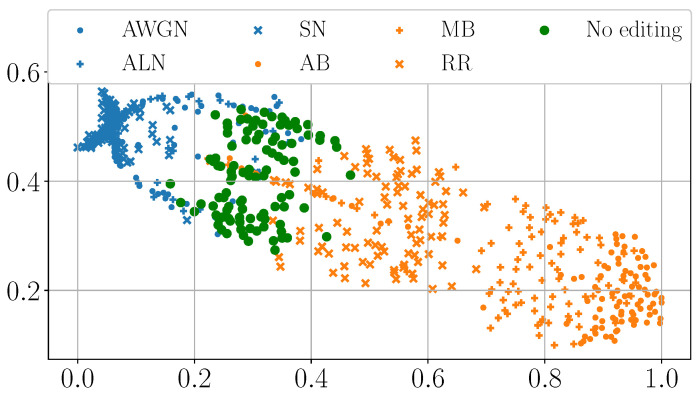
MDS visualization of the pixels belonging to the tampered-with area extracted from the DHFRs of 256×256 spliced samples.

Figure 4 and Figure 5 show the MDS visualization mentioned above. While SD2 presents tampered-with areas of various sizes, for brevity’s sake, here we report the visualization only for samples with a manipulated area of 128×128 and 256×256 pixels. However, we observed similar behaviors for the other tampering sizes. Results for the additional resolutions of 160×160, 192×192, and 224×224 are available in the code repository https://github.com/polimi-ispl/dhfr_interpretability (accessed on 22 September 2025).

Pixels spliced with noise-based editing like AWGN, ALN, and SN cluster together. Similarly, pixels spliced with blur-based operations like R&R, MB, and AB cluster together but separately from noise-based manipulations. Moreover, the “No editing” case forms a final third group separated from the others. The MDS visualization seems to confirm our previous intuition: different editing operations present in the DHFR with different brightness levels. This phenomenon is not only visible to the naked eye, but it is also confirmed at the Euclidean distance level.

As a second experiment, we also check if the appearance of the different tampered-with areas is consistent in brightness values, i.e., that noise-based manipulated areas always appear as white spots and, conversely, blur-based as black spots. Given the DHFRs extracted from SD2 through the ASAE detector, we compare them with their ground-truth tampering masks by computing the Receiving Operating Characteristic (ROC) curves and their relative Area Under the Curve (AUC).

Due to the chosen ground-truth values for the tampering mask (i.e., tampered-with pixels are equal to 1), DHFRs with brighter pixels in the tampered-with area and darker pixels outside will achieve AUC values above 0.5. Contrarily, DHFRs with darker pixels in the manipulated area will show AUC values “swapped” and below 0.5. For instance, in the DHFRs shown in Figure 3a,b the AUCs are larger than 0.5; for Figure 3c,d, the AUCs are lower than 0.5.

**Figure 6:**
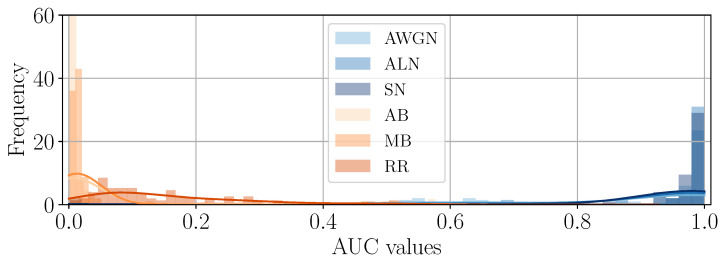
AUC values distribution of DHFRs for different editing operations on samples spliced with a 128×128 area.

**Figure 7:**
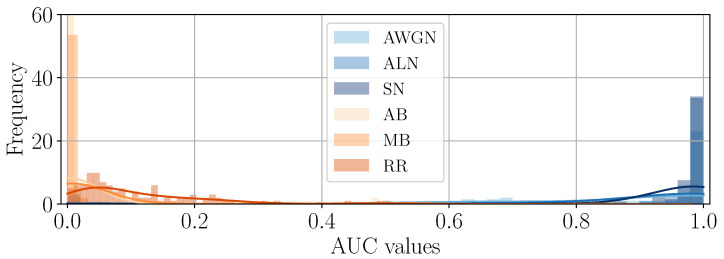
AUC values distribution of DHFRs for different editing operations on samples spliced with a 256×256 area.

Figure 6 and Figure 7 depict the distribution of AUC values for the different samples divided by editing operation. As we can see, blur-based operations lead to AUCs below 0.5, while noise-based AUCs are above this value, hence confirming the consistency of the brightness of the DHFRs across the dataset.

### 4.4. Interpretation of DHFR Appearance

As a last experiment, we want a more profound understanding of why DHFRs present such behavior to strengthen their interpretability. We conjecture the behavior in question results from a combination of the specific types of editing considered and the DHFR capabilities of capturing high-frequency details.

Specifically, we believe DHFRs offer insights into the high-frequency domain content of the sample being analyzed, including its energy. For instance, SN increases the power in the image’s high frequencies; hence, the DHFR conveys this information with a bright spot (as visible in Figure 3a). On the contrary, blurring acts as a low-pass filter, eliminating energy from the image’s high frequencies; as a result, the tampered-with area in Figure 3c appears as a dark spot due to its reduced energy content. The behaviors of R&R and AWGN are similar to the above-mentioned editing.

**Figure 8:**
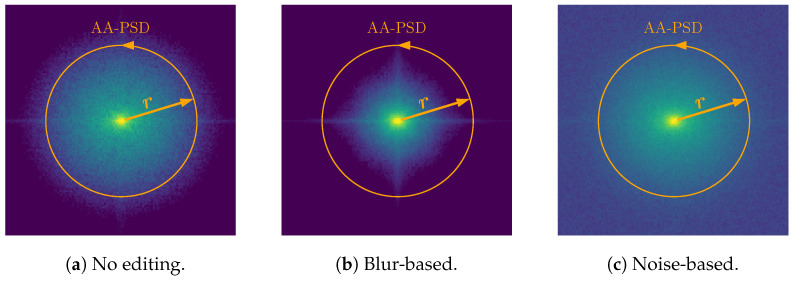
The effect of different editing operations on the average Fourier spectrum of the target region T. We also show the set of points selected for the Azimuthal Integral PSD (AA-PSD) descriptor. All spectra are centered on the DC component and plotted in the same dynamic range.

To further validate our hypothesis, we analyze the Fourier spectrum of the tampered-with areas of the different spliced samples. We divide the spliced images into three categories, namely “No editing”, “Blur-based” (AB, MB, R&R), and “Noise-based” (AWGN, ALN, SN). We then average the spectra from 25 target pixel regions T of each editing category. Figure 8 show our results. As we can easily inspect, blur-based edited images, on average, present lower energy in the high-frequency range, while noise-based editing operations have more widespread energy at all frequencies with respect to the average “No editing” spectrum.

Given the considerations relative to the spectrum of the tampered-with areas, we deepen our investigations by computing a specific scalar feature related to their Power Spectral Density (PSD). Let us define the PSD of a target pixel region T as P, represented over the spatial-frequency coordinates $w_{x},w_{y}$ as a 2D matrix of size X×Y. We define a scalar PSD integral feature as [45]

(4) $$f_{X,Y}=\sum_{w_{x}\in X}\sum_{w_{y}\in Y}P\left(w_{x},w_{y}\right),$$

where X and Y define a set of points in the $w_{x}$ and $w_{y}$ coordinates of P, respectively.

After shifting the spectrum matrix so that the DC component is at the center of P, we analyze the AA-PSD feature, which is a commonly used descriptor in the forensic literature [45,46]. To compute the AA-PSD feature, in (4) the set of points defined by X and Y should draw a circle with a specific radius *r* from the origin. To clarify, Figure 8 represents such points. By concatenating the features computed over *R* different radii, we obtain the feature vector $f_{\text{AA-PSD}}=\left[f_{X_{1},Y_{1}},\;\cdots ,\;f_{X_{R},Y_{R}}\right]$.

**Figure 9:**
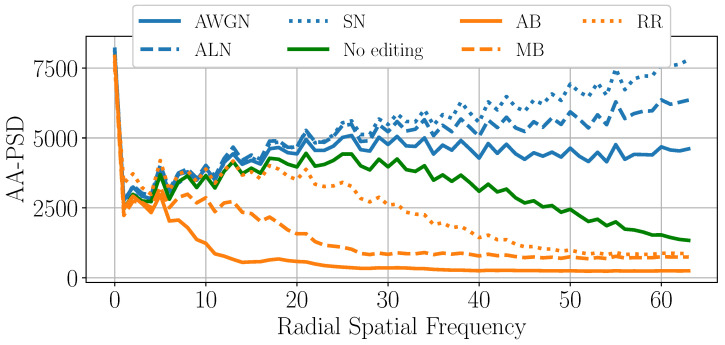
The mean AA-PSD vector extracted from the pixels belonging to the tampered-with areas of 128×128 spliced samples.

**Figure 10:**
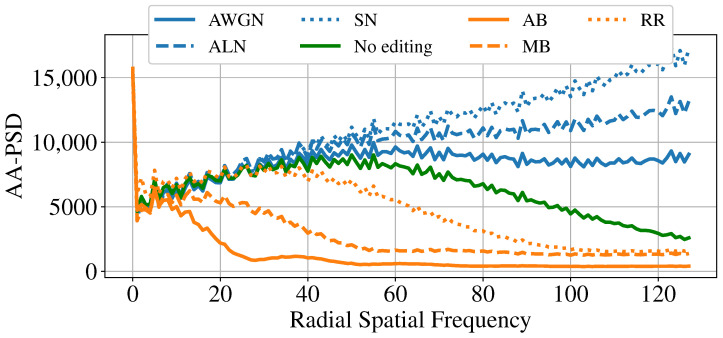
The mean AA-PSD vector extracted from the pixels belonging to the tampered-with areas of 256×256 spliced samples.

Figure 9 and Figure 10 report the average $f_{\text{AA-PSD}}$ vector for all the spliced areas of SD2, differentiated again by the editing operation applied to them. To simplify, as performed for the MDS plot, we only consider spliced samples with a target area of 128×128 and 256×256 pixels. As we can inspect, the results confirm our previous hypothesis: noise-based manipulations (blue lines) present on average a higher power content in high-frequency ranges with respect to non-edited spliced areas (green line), while blur-based edits (orange lines) show a lower power content.

Table 2 and Table 3 report a numerical quantification of this insight. In particular, we averaged the content of the AA-PSD vectors into three separate frequency ranges, namely low, medium, and high. As the reader can quickly inspect, in the high-frequency range, the PSD is considerably lower for blur-based editing, while it is considerably higher in noise-based attacks.

Please note that all the experiments conducted in this work are based on real SAR amplitude images that have been manipulated with realistic editing operations, simulating potential attacks by a malicious user. However, one limitation of the proposed study is the lack of experiments on real-world examples of locally manipulated SAR data. We believe the reliability of the SAR detector proposed in [9] and the insights provided by our interpretability analysis would be further strengthened if publicly accessible manipulated data were available.

## 5. Conclusions

In this paper, we investigated the interpretability properties of Deep High-Frequency Residuals (DHFRs) in the context of amplitude SAR image splicing localization. We linked the appearance of the DHFR in terms of brightness to its ability to represent the PSD of the image under analysis. This behavior is consistent for operations that produce similar modifications to the tampered-with areas in the high-frequency ranges of the Fourier domain.

While the explainability and interpretability flaws of NNs are well justified, we think our results highlight how the well-known combination of forensic footprints and data-driven solutions already guarantees a certain degree of interpretability. In light of the limitations of explainability techniques [6], we believe our results can be extremely valuable for forensic analysts. A simple inspection of a DHFR gives practitioners a rough estimate of what kind of operations have been executed on the image, gaining more insight into its history and more confidence in using data-driven tools.

We are confident that the present study offers a valuable contribution to the forensic community, by fostering the development of more interpretable detectors and advancing towards greater explainability of results. In particular, as deep learning now enables increasingly powerful detectors, one of the most pressing challenges is no longer solely to reach very high accuracy but rather to obtain reliable and interpretable results that can also withstand examination in environments with strong reliability requirements, e.g., law enforcement investigations.

Future works will focus on verifying our results on other data modalities like standard digital pictures and with more advanced editing operations that are known to leave artifacts in the high-frequency range, e.g., GAN and diffusion models’ image generation [46,47,48].

## Figures and Tables

**Table 1:** State-of-the-art techniques relying on DHFRs, their applications, and modality of analysis.

Detector	Task	Modality
Bonettini [36]	PRNU anonymization	Natural images
Noiseprint [10]	Image splicing localization	Natural images
Noiseprint++ [11]	Image splicing localization and detection	Natural images
ASAE [9]	Image splicing localization	SAR
SatNoiseprint [37]	Image splicing localization	Satellite RGB

**Table 2:** AA-PSD average content divided into low-, middle-, and high-frequency ranges for samples with a 128 × 128 spliced area. Similarly to the other plots, orange is associated with blur-based operations, while blue is associated with noise-based editing. In the high-frequency ranges, we use the color red to show lower power content than the “No editing” scenario; green shows higher content instead.

Operation	Low Frequencies	Medium Frequencies	High Frequencies
No editing	3731	3888	2132
AB	1725	346	247
MB	2708	956	732
RR	3828	2527	971
AWGN	4014	4741	4449
ALN	4147	5273	5778
SN	3980	5555	6817

**Table 3:** AA-PSD average content divided into low-, middle-, and high-frequency ranges for samples with a 256 × 256 spliced area. Similarly to other plots, orange is associated with blur-based operations, while blue with noise-based editing. In the high-frequency range, we use the color red to show a lower power content than the “No editing” scenario; green shows higher content instead.

Operation	Low Frequencies	Medium Frequencies	High Frequencies
No editing	7400	7755	4166
AB	3016	560	394
MB	5137	1774	1334
RR	7518	5070	1782
AWGN	7353	9093	8559
ALN	7797	10,411	11,694
SN	7596	11,391	14,687

## Data Availability

Data are contained within the article.
